# Lateral humeral condyle combined with ulnar olecranon fractures in pediatric patients: A retrospective study of treatment and outcomes

**DOI:** 10.1097/MD.0000000000049194

**Published:** 2026-06-05

**Authors:** Bangxian Dou, Huiling Tian, Xiaobo Jing, Xuedong Li

**Affiliations:** a Department of Orthopedic, Zhengzhou Orthopedic Hospital, Zhengzhou, Henan, China; b College of Pharmacy (Basic Medical Sciences), Zhengzhou Railway Vocational & Technical College, Zhengzhou, Henan, China.

**Keywords:** lateral condyle fracture of the humerus, mechanism, olecranon fracture orthopedic, pediatrics, surgical procedures

## Abstract

This study aimed to conduct a retrospective review of pediatric patients with concomitant ipsilateral lateral humeral condyle fractures and further evaluate treatment approaches and patient outcomes. Inclusion criteria: age 4 to 14 years with the diagnosis of ipsilateral lateral humeral condyle and ulnar olecranon fractures, no history of fractures, and follow-up of at least 24 months. Excluded patients: patients with open fractures, history of elbow joint surgery, and associated neurovascular injury. Surgical methods include closed reduction and percutaneous pinning or open reduction and internal fixation with/or tension-band wiring. We measured clinical and radiographic evaluation. A total of 62 patients (22 girls and 40 boys) between January 2018 and January 2022 were included in this retrospective study. The average follow-up time was 29.2 ± 3.8 months. At the final follow-up, there were no cases of cubitus valgus deformity, nonunion, delayed union, malunion, and osteoarthritis. The postoperative average elbow flexion range was 135.1 ± 7.5°. The elbow extension range was 1.5 ± 2.3°. The average forearm pronation was 82.2 ± 3.6°. The average forearm supination was 84.1 ± 2.8°. According to the Kim score, the outcome was “excellent” in 45 cases and “good” in 17 cases. The average Mayo Elbow Performance Score was 92.5 ± 3.3 points. Reduction and internal fixation yielded satisfactory clinical outcomes in the management of these rare fractures.

## 1. Introduction

Lateral humeral condyle fracture is an internal fracture of the elbow joint, accounting for 9.6% to 22.3% of all elbow fractures.^[1]^ The majority of pediatric patients are male. Most lateral humeral condyle fractures occur in children aged 4 to 10 and are the second most common type of elbow fracture.^[1]^ These fractures typically result from falling with an extended elbow, valgus impact, or direct trauma to the elbow. Ulnar olecranon fracture is rare and accounts for 5% of all elbow fractures during skeletal growth.^[2]^

These fractures typically occur between ages 5 and 10, with the most common injury mechanism being trauma to an outstretched hand or a flexed elbow. Ulnar olecranon fractures often happen alongside additional fractures of the radial head or the distal humerus. The mechanisms of injury for lateral humeral condyle and ulnar olecranon fractures are frequently similar, such as a fall onto an outstretched arm.^[1,3]^ Although similar mechanisms of injury suggest both fractures result from the same trauma, few studies have reported such injury patterns.^[4,5]^ The incidence rate of this combination of fractures is currently unclear, but it is occasionally observed in clinical practice.

Traditionally, cast treatment is the preferred option for fractures with minor displacement within 2 mm.^[3,6]^ However, for displaced fractures, open reduction and internal fixation (ORIF) are preferred. Common treatments include cast, closed reduction and percutaneous pinning (CRPP) with K-wires, as well as ORIF with or without tension-band wiring (TBW).^[7]^ However, each surgical method has its advantages and disadvantages. No consensus has been reached regarding the best procedure to achieve satisfactory results. The recommended treatment for a lateral humeral condyle fracture with an ipsilateral olecranon fracture remains controversial.

This study aimed to perform a retrospective review of consecutive ipsilateral lateral humeral condyle and ulnar olecranon fractures to evaluate treatment approaches and patient outcomes.

## 2. Materials and methods

Inclusion criteria: age 4 to 14 years with the diagnosis of ipsilateral lateral humeral condyle and ulnar olecranon fractures, no history of fractures, and follow-up of at least 24 months. Excluded patients: patients with open fractures, a history of elbow joint surgery, and associated neurovascular injury.

All patients had routine anteroposterior and lateral radiographs of the elbow. A classification system for lateral humeral condyle fracture was used according to the Song type.^[8]^ Ulnar olecranon fracture was classified according to the Mayo type.^[9]^ For lateral humeral condyle fracture, CRPP or ORIF with K-wires was performed for fixation. For olecranon fracture, CRPP with K-wires was performed for Mayo type I fracture, while K-wires or TBW was used for Mayo type II fracture. This study was approved by the Medical Research Ethics Board of Zhengzhou Orthopedic Hospital (No. 2025KY07401).

### 2.1. Surgical technique

Under general anesthesia, patients were positioned supine. For lateral humeral condyle fractures, CRPP was attempted under fluoroscopic guidance. Two parallel K-wires were inserted percutaneously from the lateral condyle. After confirming adequate stability on X-ray, the K-wires were cut short and bent within the soft tissues. For patients who failed closed reduction, ORIF was performed. We used the Kocher lateral elbow approach to expose the humeral lateral condyle, identified and removed the surrounding hematoma and remnants that might impede the fracture reduction, and carefully dissected without damaging the blood supply to the distal humerus or injuring the radial nerve bundle. Fractures were anatomically reduced under direct visualization. Two or 3 parallel K-wires were used to fix the fracture from the tip of the lateral condyle into the humerus, and successful reduction was confirmed by fluoroscopy.

**Figure 1.**
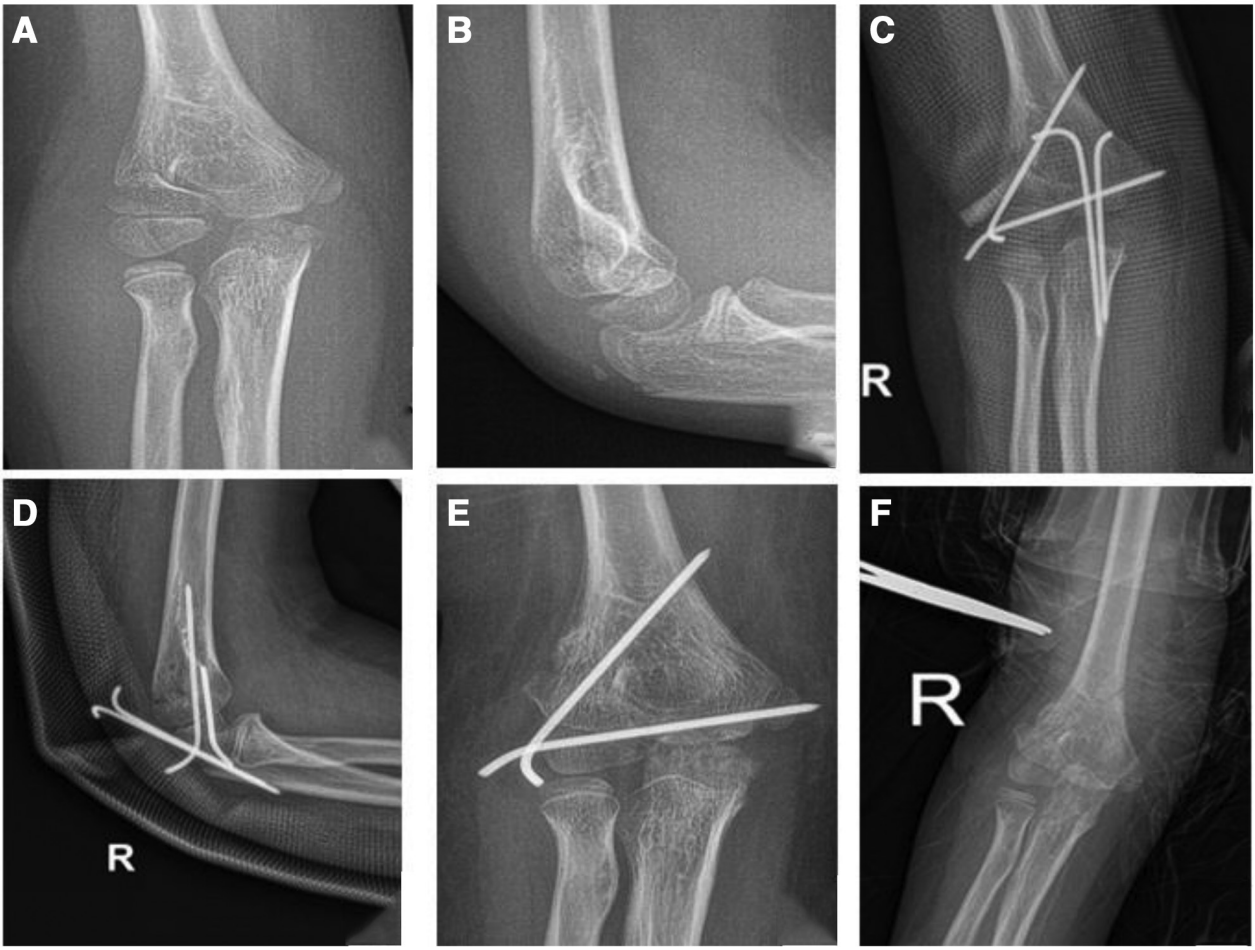
(A), (B) X-rays of a 9-year-old boy with a Song type 3 lateral condyle fracture and associated Mayo type I olecranon fracture. (C), (D) X-rays displayed with ORIF and CRPP. (E), (F) The removal results of K-wires. CRPP = closed reduction and percutaneous pinning, ORIF = open reduction and internal fixation.

For ulnar olecranon fractures, CRPP was attempted under X-ray guidance. Two parallel K-wires were inserted percutaneously from the apex of the olecranon and placed parallel to the ulnar axis. For patients who failed closed reduction for ulnar olecranon fractures, ORIF was performed. The Langenbeck posterior elbow approach was used to expose the ulnar olecranon, remove the hematoma, and anatomically reduce fractures under direct vision. K-wires were passed longitudinally from the tip of the olecranon into the distal part of the ulna, close to the articular surface in front of the ulnar olecranon (Fig. 1). For unstable comminuted fractures, TBW was employed to augment fixation stability.

Reduction stability was assessed using fluoroscopy by rotating the forearm from pronation to supination and moving the elbow from full flexion to extension. All surgeries were performed by surgeons with more than 5 years of experience in pediatric orthopedics. No additional procedures for repairing or reconstructing the ligament were performed in any of the surgeries. Bone graft was unnecessary, according to the surgeon’s experience.

### 2.2. Postoperative management

The elbow joint was immobilized at approximately 45° of flexion with the forearm in a neutral position after surgery. About 3 to 6 weeks post-operation, the anteroposterior and lateral X-ray images of the elbow were reviewed. The cast and K-wires outside the skin were removed once fracture healing was confirmed. Active rehabilitation was encouraged. Periodic reexaminations were performed to monitor for potential radiological issues and functional recovery.

### 2.3. Clinical evaluation

Perioperative data, including operation duration, bony union, and complications, were recorded. Bony union was defined as an X-ray showing blurred bone fracture lines and continuous callus passing through the fracture line. The functional outcome was assessed for all patients by the same rehabilitation specialist. At the final follow-up, the functional result was evaluated by measuring the range of motion in terms of elbow flexion, elbow extension, forearm supination, and forearm pronation with a goniometer. The functional outcome of the elbow was assessed using the Mayo Elbow Performance Score (MEPS) and the Kim score, which were based on deformity, pain, range of motion, and function, and categorized as excellent (≥90), good (75–89 points), fair (60–74 points), and poor (<60) points.^[10,11]^

### 2.4. Radiographic evaluation

Two surgeons evaluated the imaging material to determine the malunion, defined as malalignment of the subchondral bone in the humeroulnar and radiocapitellar joints, and the presence of osteoarthritis, evaluated according to the standard proposed by Kellgren and Lawrence on X-ray, which includes osteophyte formation, narrowing of joint spaces, synovitis, and altered shape.^[12]^

### 2.5. Statistical analysis

Data analysis was performed using SPSS statistical software version 24 for Windows 7 (SPSS Inc., Chicago). Continuous variables were presented as means ± standard deviations. Statistical significance was defined as *P* < .05.

## 3. Results

Between January 2018 and January 2022, 62 patients (22 girls and 40 boys) were included in this retrospective study. The average age of children was 7.2 ± 3.2 years old. Lateral humeral condyle fracture was 10 (16.1%) type 3, 22 (35.5%) type 4, and 30 (48.4%) type 5. Ulnar olecranon fracture was 26 (41.9%) type I and 36 (58.1%) type II (Table 1).

**Table 1:** Summary of characteristics of the patients.

Characteristics	Amount
Male	40 (64.5%)
Female	22 (35.5%)
Age at surgery(yr)	7.2 ± 3.2
Type 3	10 (16.1%)
Type 4	22 (35.5%)
Type 5	30 (48.4%)
Mayo type I	26 (41.9%)
Mayo type II	36 (58.1%)

The average follow-up time was 29.2 ± 3.8 months. The average operation duration was 71.2 ± 6.7 minutes. The average time to remove external K-wires was 5.2 ± 1.6 weeks. The average union time was 4.2 ± 1.5 months. No patients experienced neural injury. Five cases of superficial surgical site infections occurred, which were resolved with dressing changes. None experienced deep infections related to osteosynthesis. K-wires loosened in 4 patients, and 7 cases developed bursitis, resolving after removal of the internal fixation. All patients had their internal fixation removed at an average of 3.3 ± 2.1 months post-surgery (Table 2).

**Table 2:** Summary of general results.

Characteristics	Amount
Follow-up time (mo)	29.2 ± 3.8
Operation duration (min)	71.2 ± 6.7
Removing outside K-wires (wk)	5.2 ± 1.6
Average union time (mo)	4.2 ± 1.5
Neural injury	0
Surgical site infections	5 (8.06%)
Steel needles loosened	4 (6.45%)
Bursitis	7 (11.3%)
Lateral overgrowth	13 (20.9%)
Internal fixation removed (mo)	3.3 ± 2.1

At the final follow-up, there were no cases of cubitus valgus deformity, nonunion, delayed union, malunion, or osteoarthritis. The postoperative average elbow flexion range was 135.1 ± 7.5°, and the extension range was 1.5 ± 2.3°. The average forearm pronation was 82.2 ± 3.6°, and supination was 84.1 ± 2.8°. According to the Kim score, the outcome was categorized as “excellent” in 45 cases and “good” in 17 cases. The average MEPS was 92.5 ± 3.3 points (Table 3).

**Table 3:** Summary of evaluation.

Characteristics	Amount
Cubitus valgus deformity	0
Nonunion	0
Delayed union	0
Malunion	0
Osteoarthritis	0
Elbow flexion range (°)	135.1 ± 7.5
Extension range (°)	1.5 ± 2.3
Forearm pronation (°)	82.2 ± 3.6
Forearm supination (°)	84.1 ± 2.8
MEPS	92.5 ± 3.3
Kim score, excellent	45
Kim score, good	17

MEPS = Mayo Elbow Performance Score.

## 4. Discussion

In our study, K-wires for ipsilateral lateral humeral condyle and ulnar olecranon fractures in pediatric patients achieved satisfactory clinical results. Kai described a surgical approach involving olecranon osteotomy, where K-wires were placed across the lateral condyle fracture site in a divergent manner, and a tension-band wire construct was applied across the olecranon fracture.^[5]^ Shuai retrospectively analyzed 19 patients diagnosed with combined fractures of the lateral humeral condyle and ipsilateral ulnar olecranon. Lateral humeral condyle fractures were treated with ORIF with bioabsorbable pins. Ulnar olecranon fractures were managed with CRPP with K-wires for Mayo type IA fractures and a locking plate for Mayo type IIA fractures. The average follow-up period was 33 months. Results showed that all patients had MEPS scores over 90 and achieved good to excellent outcomes without complications.^[13]^ Occasional mention has been made of these fractures in some other case series; the lack of clinical details prevents us from analyzing them effectively.^[4,14]^ Reviewed treatments for combined fractures of the lateral condyle of the humerus and the ipsilateral olecranon. Surgical management is the next step-up surgical option when closed reduction fails to restore the fracture to an anatomic position. A consensus has never been reached on a perfect protocol to achieve satisfactory results. Screws and K-wires are commonly used for fixing the rotational fragment of the lateral condyle. TBW has been regarded as the gold standard for olecranon fractures because of its effective fixation methods and excellent results. Studies show that screw fixation has disadvantages, such as difficulty in operation, low fault tolerance, and potential impact on the growth of epiphyseal or secondary ossification centers.^[15]^ Therefore, in our report, we used K-wires instead of screws to fix humeral lateral condylar fractures. Fortunately, the results of the current method indicate it is effective, and no serious complications occurred.

The data from our study also suggested that our operation method was generally successful. A total of 62 patients completed the operation. No cases of cubitus valgus deformity, nonunion, delayed union, malunion, or osteoarthritis occurred. Forty-five cases were rated as “excellent” and 17 cases as “good.” All patients achieved a satisfactory quality of life. This might be due to the better remodeling ability in young children and optimal surgical management.

Lateral humeral condyle fractures are typically a result of playground activities and sports, and frequently occur in the summer. The most common trauma mechanism resulting in a lateral humeral condyle fracture is falling on an outstretched arm. Olecranon fractures account for 5% of all pediatric elbow fractures. The common mechanism of stress olecranon fractures involves levering of the olecranon within the olecranon fossa.^[16,17]^ Kai reported that a fall on an outstretched hand from a height or a fall with direct impact on a flexed elbow resulted in lateral humeral condyle fractures combined with ulnar olecranon fractures.^[5]^ Unfortunately, the specifics of the injury mechanisms were unclear. Shuai considered falling with an outstretched arm, wrist fully supinated, elbow joint in varus, and stress olecranon fracture as common trauma mechanisms.^[13]^ However, the chronological sequence of injury was not fully clarified. This olecranon avulsion fracture may be missed because radiographs are difficult to interpret due to the radiolucency of the cartilaginous portion and the gradual appearance of multiple ossification centers. We need to pay more attention when conducting clinical diagnosis.

We encountered some limitations in analyzing our results. First, there were inherent limitations related to the retrospective nature of our study. Second, the sample size was relatively small, and there was an absence of comparative statistical analysis in this study.

In summary, reduction and internal fixation represent a valuable adjunct in the management of this uncommon problem. We believe the proposed treatment algorithm can serve as a guide for future cases.

## Author contributions

**Conceptualization:** Xuedong Li, Xiaobo Jing.

**Project administration:** Bangxian Dou.

**Data curation:** Xiaobo Jing, Xuedong Li.

**Investigation:** Xiaobo Jing.

**Methodology:** Xiaobo Jing.

**Resources:** Xiaobo Jing.

**Supervision:** Xuedong Li.

**Validation:** Xuedong Li.

**Writing – original draft:** Bangxian Dou, Huiling Tian, Xuedong Li.

**Writing – review & editing:** Huiling Tian, Xuedong Li.
